# Prognostic impact of low *versus* high inferior mesenteric artery ligation in rectosigmoid cancer: individual patient data meta-analysis of randomized trials

**DOI:** 10.1093/bjsopen/zrag120

**Published:** 2026-09-11

**Authors:** Alberto Aiolfi, Francesco Cammarata, Emanuele Rausa, Gianluca Bonitta, Antonio Biondi, Luigi Bonavina, Davide Bona

**Affiliations:** Division of General Surgery, IRCCS Ospedale Galeazzi—Sant’Ambrogio, Milan, Italy; Department of Biomedical and Biotechnological Sciences, University of Catania, Catania, Italy; Division of General Surgery, IRCCS Ospedale Galeazzi—Sant’Ambrogio, Milan, Italy; Unit of Hereditary Digestive Tumors, Fondazione IRCCS—National Cancer Institute, Milan, Italy; Division of General Surgery, IRCCS Ospedale Galeazzi—Sant’Ambrogio, Milan, Italy; Surgical Division, Department of General Surgery and Medical Surgical Specialties, G. Rodolico Hospital, University of Catania, Catania, Italy; Department of Pharmacy, Health and Nutrition Sciences, Azienda Ospedaliera di Cosenza, Division of General and Foregut Surgery, University of Calabria, Cosenza, Italy; Division of General Surgery, IRCCS Ospedale Galeazzi—Sant’Ambrogio, Milan, Italy; Department of Biomedical Science for Health, University of Milan, Milan, Italy

**Keywords:** rectal cancer, sigmoid cancer, survival

## Abstract

**Background:**

The optimal level of inferior mesenteric artery ligation in rectosigmoid cancer surgery remains a matter of debate. This study evaluated the prognostic impact of low ligation (LL) *versus* high ligation (HL) after rectal or sigmoid cancer resection.

**Methods:**

A reconstructed individual patient data meta-analysis of randomized clinical trials (RCTs) was performed using restricted mean survival time difference (RMSTD) estimation. PubMed, MEDLINE, Scopus, Web of Science, ClinicalTrials.gov, Cochrane Central Library, and Google Scholar were searched. Primary outcomes were 5-year overall (OS) and disease-free survival (DFS). The RMSTD, risk ratio (RR), standardized mean difference (SMD), and 95% confidence interval (c.i.) were used as pooled effect size measures. The certainty of evidence was categorized with the GRADE framework.

**Results:**

Overall, 1152 patients from five RCTs were included; 34% underwent LL. Baseline demographics and pathological tumour stage were balanced among the LL and HL groups. At the 60-month follow-up, the OS and DFS estimates for LL *versus* HL were 1.40 months (95% c.i. −0.18 to 2.98; moderate level of certainty) and 0.71 months (95% c.i. −1.55 to 3.03; moderate level of certainty). Anastomotic leak (RR 0.71), overall complications (RR 0.79), total number of lymph nodes retrieved (SMD 0.01), and operating time (SMD 0.08) were comparable.

**Conclusion:**

This meta-analysis did not detect a statistically significant difference in 5-year OS and DFS between LL and HL in patients with rectosigmoid cancer.

## Teaser

The level of inferior mesenteric artery (IMA) ligation during rectosigmoid cancer surgery remains a matter of debated. Surgeons generally choose between high ligation (HL) at the IMA’s origin from the aorta or low ligation (LL) distal to the left colic artery. The impact of LL on long-term outcomes remains controversial. This study assessed the prognostic impact of LL *versus* HL after sigmoid or rectal resection for cancer. The study provides high-certainty evidence demonstrating comparable oncological outcomes for LL *versus* HL in the treatment of rectosigmoid cancers. Although not statistically significant, LL was associated with a clinical trend towards a mean 5-year overall survival improvement of 1.4 months compared with HL.

## Introduction

The optimal level of inferior mesenteric artery (IMA) ligation during rectal and sigmoid cancer surgery remains a subject of ongoing clinical debate. Surgical options include high ligation (HL) at the aortic origin of the IMA and low ligation (LL) distal to the left colic artery^1,2^. Although ensuring oncological clearance is paramount, the ligation level may also affect anastomotic integrity, vascular perfusion, and the risk of nerve injury^3,4^. HL has historically been preferred for facilitating extensive lymphadenectomy at the root of the IMA (station no. 253) and providing sufficient bowel length for a tension-free anastomosis^5,6^. Nonetheless, this method may compromise blood supply to the colonic stump, increasing the likelihood of anastomotic leakage (AL)^7^. In contrast, LL preserves the left colic artery while permitting adequate mesocolic excision, which can enhance perfusion to the anastomosis, reduce ischaemia-related leakage, and minimize the risk of iatrogenic autonomic nerve damage that could affect urinary and sexual function^8–12^. However, questions regarding the oncological adequacy of LL persist, particularly with respect to lymph node harvest and long-term survival. The effect of the level of ligation on long-term survival remains controversial, with some studies suggesting benefit for HL, whereas others indicate no significant effect^13–15^.

The purpose of this study was to assess the effect of HL *versus* LL on long-term survival following sigmoid or rectal resection for cancer. Restricted mean survival time difference (RMSTD) and a reconstructed individual patient data (IPD) meta-analysis were used to overcome limitations found in previous meta-analyses that typically used hazard ratios (HRs) or odds ratios (ORs)^2,15^. This methodology offers greater clinical interpretability than HRs and ORs, which may be prone to misinterpretation due to the assumption of constant risk throughout the follow-up period.

## Methods

A systematic review was performed following the PRISMA guidelines^16^. Ethics approval for the study was not required. The PubMed, MEDLINE, Scopus, Web of Science, ClinicalTrials.gov, Cochrane Central Register of Controlled Trials, and Google Scholar databases were searched. The literature search was conducted up to 15 February 2026 and included studies published from 1 January 2000 onwards. A combination of the following MeSH terms was used: (Rectal Neoplasms (MeSH) OR rectal cancer OR rectal carcinoma OR rectal tumour OR rectal tumor) AND (Sigmoid Neoplasms (MeSH) OR sigmoid cancer OR sigmoid carcinoma OR sigmoid tumour OR sigmoid tumor) AND (Survival Rate (MeSH) OR Mortality (MeSH) OR overall survival OR OS OR survival rate OR mortality) AND (Disease-Free Survival (MeSH) OR disease-free survival OR DFS OR recurrence-free survival), as detailed in *Appendix S1*. All titles were screened, suitable abstracts were retrieved, and the reference lists of each article were appraised.

The study was registered with PROSPERO (CRD420261280857).

### Eligibility criteria

RCTs reporting long-term Kaplan-Meier survival curves for HL *versus* LL in the setting of rectosigmoid cancer were eligible for inclusion in this study. If two or more articles were published by the same institution or study group, or used the same data set, the articles with the longest follow-up or the largest sample size were included. In the event of duplicate studies, the most recent and complete reports were included. Articles not published in English, studies with non-comparative analysis of HL *versus* LL, studies reporting mixed data including other surgical approaches or reporting aggregate data for colonic cancer, studies not reporting the *a priori* defined primary outcome or not reporting Kaplan–Meier curves, and studies with fewer than 10 patients per study arm were excluded.

### Data extraction

The following data were extracted for each of the studies: authors, year of publication, country, study design, number of patients, sex, age, body mass index (BMI), American Society of Anesthesiologists (ASA) grade, co-morbidities, tumour characteristics, surgical approach, anastomotic technique (hand-sewn *versus* stapled), postoperative outcomes, duration of follow-up, and survival. All data were independently collected by three authors (A.A., F.C., E.R.) and matched at the end of the evaluation procedure. A fourth author (L.B.) revised the database and clarified inconsistencies.

### Outcome of interest and definition

The primary outcomes were 5-year overall survival (OS) and disease-free survival (DFS). OS was defined as the time from surgery to the last known follow-up or death. DFS was defined as time from surgery to tumour recurrence (local or distant). Secondary outcomes included the total number of lymph nodes harvested, AL, operation time, intraoperative blood loss, and overall complications defined as per reporting articles. Data on OS and DFS were extracted on the basis of the reported Kaplan–Meier survival curves.

### Quality assessment and assessment of certainty of evidence

Three authors (A.A., F.C., E.R.) independently assessed the methodological quality of the selected trials using the Cochrane Risk of Bias tool 2^17^. This tool evaluates five domains: bias of randomization; allocation concealment; bias due to missing outcomes; bias in the measurements of outcomes; and bias in selection of the reported results. Thus, each RCT was graded as having a low risk of bias, some concerns, or a high risk of bias^18^. Disagreements were resolved by discussion. The quality of the evidence across studies was appraised using the GRADE approach^19^. GRADE evidence profiles were developed using GRADEpro (https://www.gradepro.org) to establish the certainty of evidence for each comparison and outcome. The certainty of evidence was determined based on factors such as bias across studies, inconsistency, indirectness, imprecision, publication bias, and other relevant parameters^20^.

### Statistical analysis

The results of the systematic review were summarized qualitatively into a frequentist meta-analysis of RMSTD^21,22^. The individual patient time-to-event data were reconstructed from Kaplan–Meier curves. The curves were digitalized using Get Data Graph Digitizer software (http://getdata-graph-digitizer.com). The pooled RMSTD was calculated using a random-effect multivariate meta-analysis borrowing strength across time points with within-trial covariance^23^. In addition, using IPD, a flexible hazard-based regression model was created with the inclusion of a normally distributed random intercept. Specifically, the baseline hazard was modelled as the exponential of a degree-3 B-spline with no interior knots. Model selection was driven according to Akaike’s information criterion^24^. The time-dependent effects of surgical treatment were parameterized as interaction terms between the surgical treatment and baseline hazard and statistically tested using the likelihood ratio test. The hazard functions plot was created using marginal prediction^25^. Two-sided *P* < 0.05 was considered statistically significant and 95% confidence intervals were computed. Statistical analyses were performed using R version 4.5.1 (R Foundation for Statistical Computing, Vienna, Austria, http://www.R-project.org).

## Results

### Systematic review

**Figure 1:**
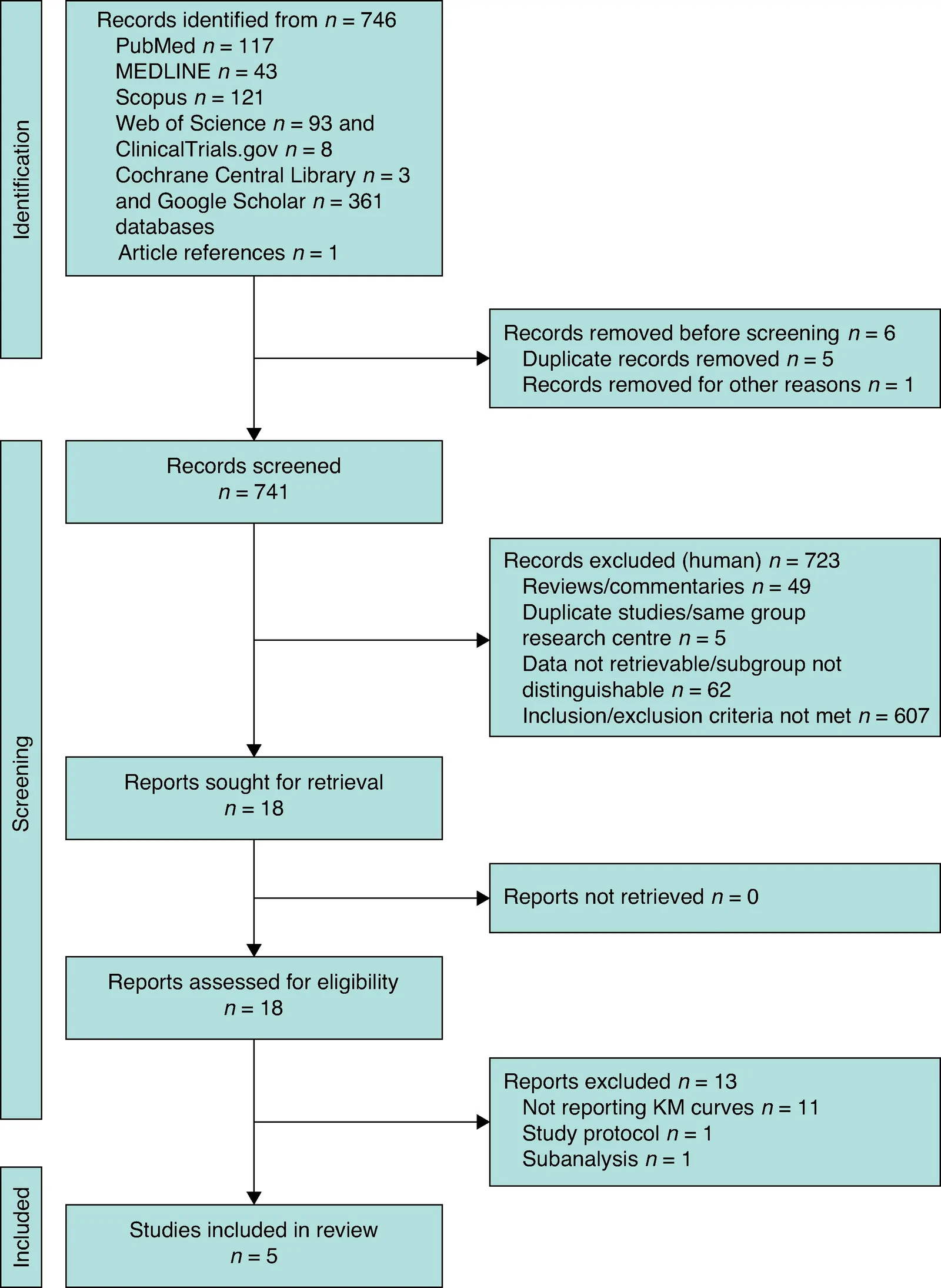
PRISMA diagram Records were identified from the PubMed, MEDLINE, Scopus, Web of Science, ClinicalTrials.gov, and the Cochrane Central Register of Controlled Trials databases; conference abstracts, book chapters, conference posters, letters, editorials, or articles on unrelated topics were excluded. KM, Kaplan–Meier.

The selection flow chart is shown in *Fig. 1*. Overall, 746 publications were identified, and 741 titles were screened after the removal of duplicates, with 18 full-text articles identified as possibly relevant. After evaluation, five RCTs^26–30^ met the inclusion/exclusion criteria and were incorporated in the quantitative analysis. All included papers were RCTs: three single centre^26,28,29^ and two multicentre^27,30^ RCTs. Randomization methods were described in one study^30^, and surgeon eligibility criteria were specified in two^27,30^. The trial by Akagi *et al*.^27^ involved an unequal allocation ratio, because randomization was not performed on a 1 : 1 basis. Surgical quality control measures were reported by only one study^27^; data regarding blinding of outcomes assessors or statisticians were not provided by any study. Two studies^27,30^ included a formal power analysis, with one^27^ identified as underpowered. None of the studies indicated whether their analysis followed an intention-to-treat or per-protocol approach (*Table S1*). Of the studies included, four^26–29^ were identified as presenting some concerns regarding bias, whereas one^30^ was assessed as having a low risk of bias (*Fig. S1*).

Overall, 1152 patients with rectosigmoid cancer undergoing resection were included for quantitative analysis (*Table 1*). LL was performed in 34% (392) of patients. Included patients ranged in age from 28 to 86 years, and most were men (60.3%). The preoperative BMI was reported in three studies^28–30^, while the ASA grade was reported in two^26,30^. Tumour histology and tumour location were defined in all papers, stratified in lower rectum, middle rectum, upper rectum, and sigmoid colon. Pathological tumours were staged according to the 7th and 8th editions of the American Joint Commission on Cancer, with proportions as follows: stage 0–I, 19.4%; stage II, 49.3%; stage III, 30.7%; and stage IV, 0.6%. Minimally invasive surgery was performed in 57.7% of patients. Baseline demographics and pathological stage were well balanced among the two groups. Data on protective diverting stoma were heterogeneous and non-randomized depending on operating surgeon preference, lower rectal cancer location, preoperative chemoradiation, suspicion of poor anastomosis, and positive air leak during surgery. Data on neoadjuvant treatments were reported in four studies^26,28–30^, with 28.2% of patients undergoing preoperative chemotherapy or chemoradiation therapy. Adjuvant treatment was described in two trials^26,30^. None of the studies reported in-hospital mortality.

**Table 1:** Demographic, clinical, and operative data for patients undergoing surgery for rectal or sigmoid cancer with HL or LL of the inferior mesenteric artery

Study	Country	Study period	Surgical procedure	No. of patients	Age>* (years)	Sex (*n*)		BMI* (kg/m^2^)	Tumour location (*n*) (L/M/U/Sig)	Histology (ADC)	Pathological stage (*n*)	NeoAD	AD
						Male	Female						
Matsuda *et al*.^26^ (2017)	Japan	2008–2011	HL	51	69	33	36	NR	15/20/16/0	51	I, 9; II, 15; III, 3; IV, 4	2	29
			LL	49	67	34	33		15/21/13/0	49	I, 1; II, 7; III, 17; 13; IV, 2	5	20
Akagi *et al*.^27^ (2020)	Japan	2009–2012	HL	496	64 (28–75)	305	191	NR	171†/325	NR	I, 0; II, 338; III, 158; IV, 0	NR	NR
			LL	135	60 (39–75)	74	61		49†/86	NR	I, 0; II, 98; III, 37; IV, 0	NR	NR
Feng *et al*.^28^ (2021)	China	2016–2018	HL	47	60.5(10.2)	26	21	22.6(2.3)	NR†	47	I, 21; II, 12; III, 14; IV, 0	0	NR
			LL	48	59.8(9)	24	24	22.9(2.7)		48	I, 25; II, 13; III, 10; IV, 0		
Kruszewski *et al*.^29^ (2021)	Poland	2010–2016	HL	65	64(9)	37	28	27(4)	NR†	65	I, 32; II, 14; III, 19; IV, 0	42	NR
			LL	65	65(8.5)	34	31	27(4)		65	I, 23; II, 18; III, 24; IV, 0	43	NR
Mari *et al*.^30^ (2023)	Italy	2014–2016	HL	101	64 (34–84)	65	36	26.7(4.6)	24/62/15/0	101	I, 41; II, 22; III, 38; IV, 0	30	56
			LL	95	68 (35–86)	63	32	26.1(3.9)	33/54/8/0	95	I, 55; II, 21; III, 19; IV, 0	25	40

^*^Values are the mean(standard deviation) or median (interquartile range) unless otherwise stated. †All rectal cancers. HL, high ligation; LL, low ligation; BMI, body mass index; L, lower rectum; M, middle rectum; U, upper rectum; Sig, sigmoid colon; ADC, adenocarcinoma; NeoAD, neoadjuvant treatment (chemotherapy or chemoradiation therapy); AD, adjuvant therapy; NR, not reported.

### IPD analysis

**Figure 2:**
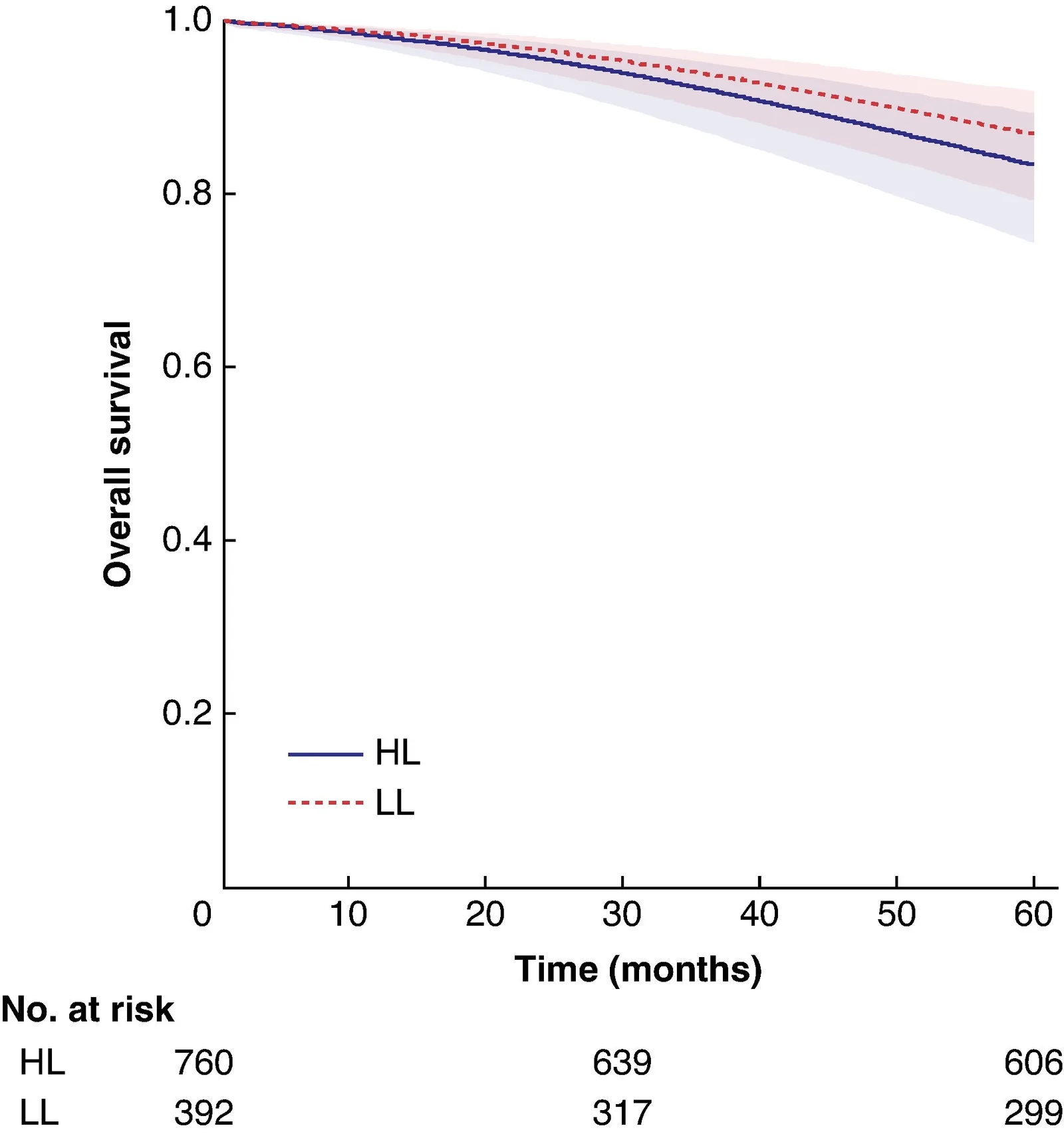
Estimated pooled overall survival for LL and HL Solid lines represent the predicted overall survival curve, with the dotted lines denoting the corresponding 95% confidence intervals. LL, low ligation; HL, high ligation.

The clinical appraisal of the OS RMSTD was based on Kaplan–Meier curves reported in all five studies (*Fig. S2*). The RMSTD and the time horizons are detailed in *Table 2*. At the 60-month follow-up, the combined effect derived from the multivariate meta-analysis and RMSTD estimation for the comparison of LL *versus* HL was 1.40 months (95% confidence interval (c.i.) −0.18 to 2.98 months; *P* = 0.084). This difference was not statistically significant. The estimated pooled OS for LL *versus* HL is shown in *Fig. 2*, demonstrating similar 5-year follow-up survival rates (87.3% *versus* 84.1%, respectively). Considering the non-proportional hazard models (*P* < 0.001), the time-varying OS HRs for LL *versus* HL were also assessed, with no significant differences detected up to the 60-month follow-up (*Fig. S3*). Minimal heterogeneity was observed (*I*^2^ = 0%; τ^2^ = 0.075; 95% c.i. 0.0 to 0.12).

**Table 2:** RMSTD for overall survival at different time points for low ligation *versus* high ligation after rectal or sigmoid cancer resection

	RMSTD (months)	*P* value
12 months	0.02 (−0.02, 0.07)	0.298
24 months	0.16 (−0.11, 0.43)	0.242
36 months	0.47 (−0.34, 1.28)	0.256
48 months	0.82 (−0.35, 2.01)	0.177
60 months	1.40 (−0.18, 2.98)	0.083

Values in parentheses are 95% confidence intervals. RMSTD, restricted mean survival time difference.

**Figure 3:**
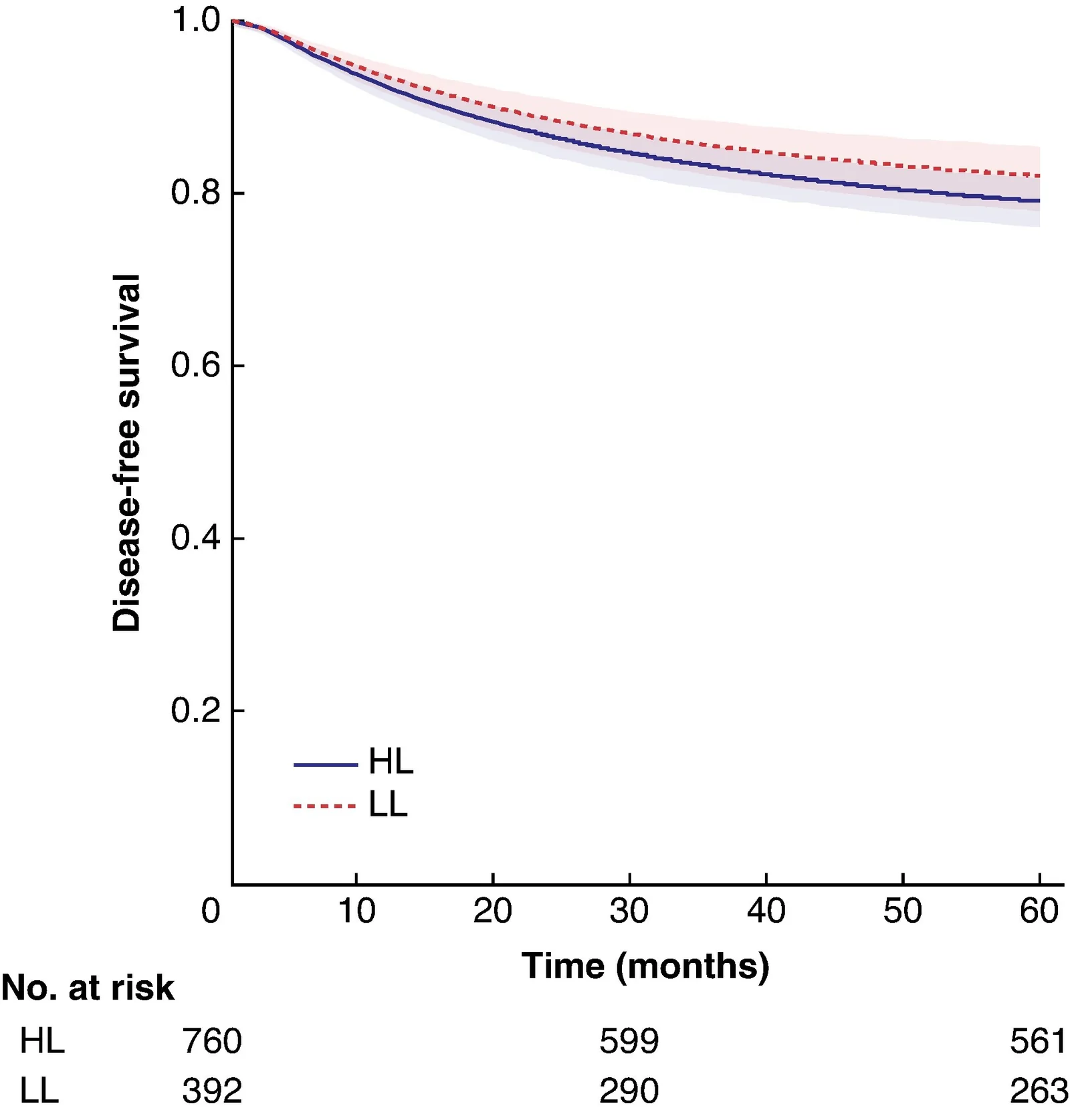
Estimated pooled disease-free survival for LL and HL Solid lines represent the predicted disease-free survival curve, with the dotted lines denoting the corresponding 95% confidence intervals. LL, low ligation; HL, high ligation.

The clinical appraisal of the DFS RMSTD was based on Kaplan–Meier curves reported in all five studies (*Fig. S4*). The RMSTD and time horizons are detailed in *Table 3*. At the 60-month follow-up, the combined effect derived from the multivariate meta-analysis and RMSTD estimation for LL *versus* HL was 0.71 months (95% c.i.. −1.55 to 3.03 months; *P* = 0.521). This difference was not statistically significant. The estimated pooled DFS for LL *versus* HL is shown in *Fig. 3*, demonstrating similar 5-year follow-up survival rates (82% *versus* 79.2%, respectively). Considering the non-proportional hazard models (*P* < 0.001), the time-varying DFS HRs for LL *versus* HL were also assessed, with no significant differences up to 60-month follow-up (*Fig. S5*). Low heterogeneity was observed (*I*^2^ = 0%; τ^2^ = 0.055; 95% c.i. 0.0–0.11). Using the GRADE tool, the certainty of evidence for 5-year OS and 5-year DFS was determined as moderate (*Table S2*).

**Table 3:** RMSTD for disease-free survival at different time points for low ligation *versus* high ligation after rectal or sigmoid cancer resection

	RMSTD (months)	*P*
12 months	0.08 (−0.03, 0.21)	0.165
24 months	0.11 (−0.46, 0.68)	0.242
36 months	0.26 (−0.85, 1.01)	0.711
48 months	0.43 (−1.26, 2.13)	0.628
60 months	0.73 (−1.55, 3.03)	0.534

Values in parentheses are 95% confidence intervals. RMSTD, restricted mean survival time difference.

### Secondary outcomes

No significant differences were found for LL *versus* HL in AL rates, overall complications, total number of lymph nodes retrieved, and operating time (*Table 4*). The sensitivity analysis showed the robustness of these findings in terms of point estimation, relative confidence intervals, and heterogeneity.

**Table 4:** Secondary outcomes after low ligation *versus* high ligation after rectal or sigmoid cancer resection

Outcomes	RR	*I* ^2^ (%)
Anastomotic leak	0.71 (0.42, 1.21)	0.0
Overall complications	0.79 (0.57, 1.09)	48.7
Operative time (minutes)	0.08* (−11.3, 11.5)	13.2
Harvested lymph nodes	0.01* (−1.47, 1.45)	56.5

Values in parentheses are 95% confidence intervals. *Standardized mean difference. RR, risk ratio; *I*^2^, heterogeneity.

## Discussion

This IPD analysis presents the latest evidence regarding the prognostic significance of LL *versus* HL during rectosigmoid cancer resection. Over the past 10 years, several meta-analyses have attempted to highlight the oncological superiority of one over another, but the topic is still debated^2,15,31,32^. Unlike previous aggregate data meta-analyses, the present study may provide a more methodologically robust and clinically informative assessment. Previous studies were based on aggregate estimates (HR or OR), inherently relying on the proportional hazard assumption with limited clinical interpretability. In contrast, the IPD approach enables reconstruction of Kaplan–Meier curves at the individual level, direct modelling of time-to-event data, formal testing of non-proportional hazards, and the use of flexible hazard-based regression models with time-varying effects. The use of RMSTD overcomes the interpretative limitations of HRs by quantifying survival benefit in absolute time units, thereby offering a clinically intuitive measure of effect across a predefined follow-up period. Furthermore, the multivariate random-effects framework incorporating within-trial covariance and spline-based baseline hazard modelling enhances statistical robustness compared with conventional pooling of summary estimates^33–35^. In this context, the reconstructed IPD analysis in this study suggests no clinically meaningful oncological differences between LL and HL, and provides a clinically interpretable assessment of long-term outcomes.

Radical resection with an adequate circumferential margin and lymphadenectomy continues to be the standard treatment for localized rectosigmoid cancers, facilitating accurate staging, improved local disease control, and optimal oncological outcomes. According to the National Comprehensive Cancer Network, a minimum of 12 lymph nodes should be harvested to determine pathological stage^36^. Other studies suggest excising higher numbers of lymph nodes, depending on disease stage, to enhance long-term survival and reduce cancer recurrence. Japanese surgeons advocate for N3 dissection based on its perceived OS and DFS benefits in patients with a tumour exhibiting local spread beyond T2 or when clinically evident nodal metastases are present^37,38^. In contrast, US guidelines generally recommend limited dissection for most rectal cancer patients due to the rarity of nodal metastases at the root of the IMA^39^. In addition, patients with central nodal metastasis have low 5-year survival rates (ranging from 0% to 40%)^26^, indicating that the resection of these lymph nodes does not significantly improve survival outcomes^40,41^. In the context of rectosigmoid cancer, numerous studies have assessed oncological outcomes associated with HL *versus* LL^26–30^. The primary rationale for HL is the excision of N3 lymph nodes located at the origin of the inferior mesenteric artery (station no. 253). In the present study, a comparable total number of harvested lymph nodes was found for LL and HL, aligning with previous research^12,42,43^ indicating that most regional nodes are situated distal to the origin of the IMA and can be adequately retrieved regardless of ligation level. This evidence suggests that appropriately performed LL ensures a lymphadenectomy comparable to that with HL. Notably, due to moderate heterogeneity (*I*^2^ = 56%) related to institutional technical variability, surgeon experience, pathology expertise, and differences in the definitions of IMA ligation levels, caution should be exercised when interpreting these findings.

Nowadays, patients with clinically suspicious lymph node metastasis at the root of the IMA seem to benefit the most from HL. The American Society of Colon and Rectal Surgeons recommends HL only when suspicious or proven apical nodes are present at the IMA root or when additional length is required for a tension-free anastomosis^44–46^. Most patients with sigmoid and rectal cancer, especially those without apical lymph node involvement or advanced T/N category, benefit most from LL of the IMA. This is particularly advantageous in patients with co-morbidities, poor vascular status, or those at higher risk of anastomotic complications. In fact, LL preserves the left colic artery, which maintains better blood supply to the anastomosis and reduces the risk of ischaemic complications^9,47^. Moreover, some studies have also reported^48^ improved postoperative defaecatory function and lower rates of low anterior resection syndrome in LL patients. In this context, older individuals are most susceptible to changes in bowel function and continence following the selection of HL *versus* LL. This susceptibility is attributed to age-related baseline pelvic floor weakness and reduced neural reserve, which amplify the impact of hypogastric plexus injury and compromised blood supply associated with HL^49–51^.

The comparison of survival outcomes between HL and LL remains a matter of debate. Slanetz and Grimson^52^ observed significantly improved 5-year survival rates in the HL group for Dukes’ B and C rectal cancer patients. Similarly, Singh *et al*.^32^ reported greater OS advantages for HL over LL in patients with IMA-positive lymph nodes (HR 0.77; 95% c.i. 0.66 to 0.89). Conversely, other trials (for example, HIGHLOW) did not reveal meaningful differences in survival between HL and LL, although their findings were limited by short follow-up periods and insufficient statistical power for survival endpoints^53^. In contrast, recent meta-analyses of RCTs indicate superior 5-year overall survival in the LL cohort^15^. In the present IPD analysis, the RMSTD assessment demonstrated no statistically significant dissimilarities for 5-year OS (1.40 months; 95% c.i. −0.18 to 2.98; *P* = 0.084) for LL *versus* HL. These findings differ from those of Sassun *et al*.^15^, who reported superior 5-year OS with LL *versus* HL (HR 0.69; *P* = 0.02). This discrepancy may be explained by several factors. HL may be associated with a higher risk of AL and overall complications^7^, which could contribute to transient immunosuppression, favour tumour progression^54^, and impair tolerance of adjuvant therapy. In the present study, the RMSTD DFS analysis concluded comparable results for LL *versus* HL (0.71 months; *P* = 0.521). This is similar to findings reported by Sassun *et al*.^15^ (HR 0.84; *P* = 0.79) and Cirocchi *et al*.^2^ (RR 0.97), who found comparable 5-year DFS for the two surgical approaches. This may be attributable to the fact that tumour recurrence is primarily influenced by the quality of total mesorectal excision and the status of surgical resection margins rather than the level of IMA ligation^48,55^. Moreover, the incidence of distant tumour recurrence, primarily in the liver and lungs, does not depend on the level at which the IMA is ligated^26^.

Despite the related heterogeneity being minimal, several factors should be considered when interpreting the findings of the present study. First, three of the five included trials were conducted at tertiary centres in Asian countries, which may restrict generalizability. Second, although all studies were RCTs, many had limitations due to their relatively small sample sizes, randomization method, blinding, and heterogeneity (for example, diverting stoma formation). Third, because most trials were powered on short-term outcomes, data on long-term survival may have been underpowered. Fourth, although neoadjuvant and adjuvant therapies were reported, details regarding their implementation and compliance remained limited. Fifth, factors such as *KRAS* or microsatellite mutations, tumour genetic profiles, enhanced recovery protocols, and the impact of short-term outcomes on survival data were not considered. Sixth, the absence of tumour stage stratification should be considered, even though previous studies found no significant differences or even better OS and DFS for stage III disease in LL patients^29^. Finally, the study by Akagi *et al*. included data on both sigmoid and rectal cancer^27^; however, previous research indicates that tumour location does not influence oncological outcomes and that the surgical approach for these cancers is methodologically and clinically consistent with rectal cancers^15,27^.

Several limitations merit consideration. Notwithstanding the rigorous application of the statistical methodology, the IPD meta-analysis based on reconstructed Kaplan–Meier curves remains susceptible to possible measurement error. Baseline heterogeneity among patients, including variations in co-morbidities, vascular anatomical variations^56^, and inconsistencies in reporting oncological variables such as tumour grading, and adherence to adjuvant therapies may influence outcomes. In addition, slight between-study variation in the definition of IMA ligation level may represent a further source of methodological heterogeneity. Differences in perioperative multidisciplinary care, surgical techniques, surgeon experience, postoperative complications, and the genomic/biological characteristics of tumours can impact long-term survival results. Further, although all included studies were RCTs, they were underpowered regarding the primary endpoints analysed; thus, the findings may be biased. The exclusion of articles published in languages other than English may constitute an additional source of bias. Finally, functional, urinary, and sexual outcomes were not evaluated, because the primary focus was long-term survival data.

## Supplementary Material

zrag120_Supplementary_Data

## Data Availability

The data collected and analysed during this review are available from the corresponding author upon reasonable request.
