# Supplementary material for: Prognostic impact of low versus high inferior mesenteric artery ligation in rectosigmoid cancer: individual patient data meta-analysis of randomized trials
Source: BJS Open. 2026 Sep 11;10(5):zrag120. doi: 10.1093/bjsopen/zrag120 (PMC13563187; doi:10.1093/bjsopen/zrag120)
Supplement: Supplementary file 1 [file zrag120_supplementary_data.docx]

**Title.** PROGNOSTIC IMPACT OF LOW versus HIGH INFERIOR MESENTERIC ARTERY LIGATION IN RECTOSIGMOID CANCER: INDIVIDUAL PATIENT DATA META-ANALYSIS OF RANDOMIZED TRIALS

**Authors**. Alberto Aiolfi MD FACS FEBS (UGI) ^1^, Francesco Cammarata MD ^1^, Emanuele Rausa MD ^2^, Gianluca Bonitta MSc ^1^, Antonio Biondi MD ^3^, Luigi Bonavina MD FACS (Hon) ^4^ Davide Bona MD FACS ^1^

**Affiliations**

1. I.R.C.C.S. Ospedale Galeazzi – Sant’Ambrogio, Division of General Surgery, Department of Biomedical Science for Health, University of Milan, Milan, Italy
2. Unit of Hereditary Digestive Tumors, Fondazione IRCCS-National Cancer Institute, Milan, Italy.
3. G. Rodolico Hospital, Surgical Division, Department of General Surgery and Medical Surgical Specialties, University of Catania, 95131 Catania, Italy.
4. Department of Pharmacy, Health and Nutrition Sciences, Azienda Ospedaliera di Cosenza, Division of General and Foregut Surgery, University of Calabria, Cosenza, Italy.

**Corresponding author.** Alberto Aiolfi **ORCID ID** 0000-0002-7764-6075

**Supplementary Materials - Index**

| **Supplementary Methods** |  |
| --- | --- |
| NA |  |
| **Supplementary Results** |  |
| NA |  |
| **Supplementary Appendixes** |  |
| Appendix 1 – Seach Strategies pag 4-6 |  |
| **Supplementary Figures and Tables** |  |
| Supplementary figures | *pag. 7-9* |
| Supplementary tables | *pag. 10-11* |
| **References** | *NA* |
|  |  |

**Supplementary Methods**

**NA**

**Supplementary Results**

**NA**

**Supplementary Appendixes**

**Appendix 1 – Seach Strategies**

**PubMed**

(

"Rectal Neoplasms"[Mesh]

OR rectal cancer[tiab]

OR rectal carcinoma[tiab]

OR rectal tumor*[tiab]

OR rectal tumour*[tiab]

)

OR

(

"Sigmoid Neoplasms"[Mesh]

OR sigmoid cancer[tiab]

OR sigmoid carcinoma[tiab]

OR sigmoid tumor*[tiab]

OR sigmoid tumour*[tiab]

OR "sigmoid colon cancer"[tiab]

OR "sigmoid colon carcinoma"[tiab]

)

AND

(

"Survival Rate"[Mesh]

OR "Mortality"[Mesh]

OR "overall survival"[tiab]

OR OS[tiab]

OR survival[tiab]

OR mortality[tiab]

)

AND

(

"Disease-Free Survival"[Mesh]

OR "disease-free survival"[tiab]

OR DFS[tiab]

OR "recurrence-free survival"[tiab]

OR "relapse-free survival"[tiab]

)

**Scopus**

TITLE-ABS-KEY(

"rectal neoplasm*" OR

"rectal cancer*" OR

"rectal carcinoma*" OR

"rectal tumor*" OR

"rectal tumour*" OR

"sigmoid neoplasm*" OR

"sigmoid cancer*" OR

"sigmoid carcinoma*" OR

"sigmoid tumor*" OR

"sigmoid tumour*" OR

"sigmoid colon cancer*" OR

"sigmoid colon carcinoma*"

)

AND

TITLE-ABS-KEY(

survival OR

"overall survival" OR

OS OR

mortality OR

prognosis OR

prognostic OR

outcome* OR

"disease-free survival" OR

DFS OR

"recurrence-free survival" OR

"relapse-free survival" OR

"progression-free survival" OR

PFS

)

**MEDLINE**

((exp Rectal Neoplasms/ or (rectal cancer* or rectal carcinoma* or rectal tumor* or rectal tumour*).ti,ab,kf.)

or

(exp Sigmoid Neoplasms/ or (sigmoid cancer* or sigmoid carcinoma* or sigmoid tumor* or sigmoid tumour* or sigmoid colon cancer* or sigmoid colon carcinoma*).ti,ab,kf.))

and

(exp Survival Rate/ or exp Mortality/ or ("overall survival" or OS or survival rate* or survival or mortality).ti,ab,kf.)

and

(exp Disease-Free Survival/ or ("disease-free survival" or DFS or "recurrence-free survival" or "relapse-free survival").ti,ab,kf.)

**Web of Science**

TS=(

"rectal neoplasm*" OR

"rectal cancer*" OR

"rectal carcinoma*" OR

"rectal tumor*" OR

"rectal tumour*" OR

"sigmoid neoplasm*" OR

"sigmoid cancer*" OR

"sigmoid carcinoma*" OR

"sigmoid tumor*" OR

"sigmoid tumour*" OR

"sigmoid colon cancer*" OR

"sigmoid colon carcinoma*"

)

AND

TS=(

survival OR

"overall survival" OR

OS OR

mortality OR

prognosis OR

prognostic OR

outcome* OR

"disease-free survival" OR

DFS OR

"recurrence-free survival" OR

"relapse-free survival" OR

"progression-free survival" OR

PFS

)

**ClinicalTrials.gov**

(

"rectal cancer" OR

"rectal carcinoma" OR

"rectal neoplasm" OR

"rectal tumor" OR

"rectal tumour"

OR

"sigmoid cancer" OR

"sigmoid carcinoma" OR

"sigmoid neoplasm" OR

"sigmoid tumor" OR

"sigmoid tumour" OR

"sigmoid colon cancer"

)

AND

(

"overall survival" OR

survival OR

mortality OR

"survival rate"

)

AND

(

"disease-free survival" OR

DFS OR

"recurrence-free survival" OR

"relapse-free survival"

)

**Cochrane Central Library**

#1 MeSH descriptor: [Rectal Neoplasms] explode all trees

#2 MeSH descriptor: [Sigmoid Neoplasms] explode all trees

#3 (rectal cancer* OR rectal carcinoma* OR rectal tumor* OR rectal tumour*):ti,ab,kw

#4 (sigmoid cancer* OR sigmoid carcinoma* OR sigmoid tumor* OR sigmoid tumour* OR "sigmoid colon cancer*" OR "sigmoid colon carcinoma*"):ti,ab,kw

**Google Scholar**

("rectal cancer" OR "rectal carcinoma" OR "rectal neoplasm" OR

"sigmoid cancer" OR "sigmoid carcinoma" OR "sigmoid neoplasm")

("overall survival" OR mortality OR "disease-free survival" OR

"recurrence-free survival")

**Supplementary Figures and Tables**

**Figure S1**. Risk of Bias 2 assessment for the included studies. Legend. Domain 1: Randomization process. Domain 2: Deviations from the intended interventions. Domain 3: Missing outcome data. Domain 4: Measurement of the outcome. Domain 5: Selection of the reported results

**Figure S2**. Restricted mean survival time difference (RMSTD) for OS. Each color represents a single study. The pooled RMSTD with relative 95% CI using different statistical methods estimation is represented by black points (triangle, circle).

**Figure S3**. LL vs. HL OS hazard ratio variations (Y axis). Continued tracts represent the estimated pooled hazards while dotted tracts represent the 95% Confidence Interval (95% CI). Time (X axis) expressed in months.

**Figure S4**. Restricted mean survival time difference (RMSTD) for DFS. Each color represents a single study. The pooled RMSTD with relative 95% CI using different statistical methods estimation is represented by black points (triangle, circle).

**Figure S5**. LL vs. HL DFS hazard ratio variations (Y axis). Continued tracts represent the estimated pooled hazards while dotted tracts represent the 95% Confidence Interval (95% CI). Time (X axis) expressed in months.

| **Author, year** | **Trial Name and Registration** | **Study design** | **Method of randomization** | **Surgeons' eligibility** | **Surgical quality control** | **Blinding** | **Formal power analysis** | **ITT/PP** |
| --- | --- | --- | --- | --- | --- | --- | --- | --- |
| Matsuda et al., 2017 ^(27)^ | NCT00701012 | SC | nr | nr | nr | nr | nr | nr |
| Akagi et al., 2020 ^(28)^ | JCOG0404 | MC | nr | Surgeons experienced in colorectal surgery with >30 copen or lap colectomies performed | Photographs of the surgical field | nr | UP  (subgroup analysis from the original paper) | nr |
| Feng et al., 2021 ^(29)^ | ChiCTR-INR-17010336 | SC | nr | nr | nr | nr | nr | nr |
| Kruszewski et al., 2021 ^(30)^ | ISRCTN12189372 | SC | nr | nr | nr | nr | nr | nr |
| Mari et al., 2023 ^(31)^ | HIGHLOW | MC | Computerized randomization system and sealed envelopes; randomization balance for sex, and neoadjuvant treatment | Surgeons performing at least 20 laparoscopic colonic resection procedures per year during the previous 5 years | nr | nr | Y | nr |

**Supplementary Table 1**. Randomized Clinical Trials (RCTs) quality evaluation. SC single center, MC multicenter, UP under-power, Y yes, ITT: Intention-to-treat. PP: per-protocol or as-treated. Nr not reported

| **Certainty assessment** | | | | | | | **№ of patients** | | **Effect** | | **Certainty** | | **Importance** | | |
| --- | --- | --- | --- | --- | --- | --- | --- | --- | --- | --- | --- | --- | --- | --- | --- |
| **№ of studies** | **Study design** | **Risk of bias** | **Inconsistency** | **Indirectness** | **Imprecision** | **Other considerations** | **LL** | **HL** | **Relative (95% CI)** | **Absolute (95% CI)** | |  | | |  |
| **5-year overall survival (follow-up: mean 60 months)** | | | | | | | | | | | | | | | |
| 5 | RCTs | not serious | not serious | not serious | not serious | none |  |  |  | **SMD 1.40 higher** (-0.18 lower to 2.98 higher) | | ⨁⨁⨁◯ Moderate | | CRITICAL | |
| **5-year disease-free survival (follow-up: mean 60 months)** | | | | | | | | | | | | | | | |
| 5 | RCTs | not serious | not serious | not serious | not serious | none |  |  |  | **SMD 0.71 higher** (-1.55 lower to 3.03 higher) | | ⨁⨁⨁◯ Moderate | | | CRITICAL |

**Supplementary Table 2**. The quality of evidence with the GRADE methodology

**References**
